# White paper on microbial anti-cancer therapy and prevention

**DOI:** 10.1186/s40425-018-0381-3

**Published:** 2018-08-06

**Authors:** Neil S. Forbes, Robert S. Coffin, Liang Deng, Laura Evgin, Steve Fiering, Matthew Giacalone, Claudia Gravekamp, James L. Gulley, Hal Gunn, Robert M. Hoffman, Balveen Kaur, Ke Liu, Herbert Kim Lyerly, Ariel E. Marciscano, Eddie Moradian, Sheryl Ruppel, Daniel A. Saltzman, Peter J. Tattersall, Steve Thorne, Richard G. Vile, Halle Huihong Zhang, Shibin Zhou, Grant McFadden

**Affiliations:** 1 Department of Chemical Engineering, University of Massachusetts, 159 Goessmann Hall, Amherst, MA 01003 USA; 2 Replimune, Abingdon, UK; 3 Department of Medicine, Memorial Sloan Kettering Cancer Center, New York, NY 10065 USA; 4 Mayo Clinic, Rochester, USA; 5 Geisel School of Medicine at Dartmouth, Hanover, USA; 6 Vaxiion Therapeutics, San Diego, USA; 7 Albert Einstein College of Medicine, Bronx, USA; 8 National Cancer Institute, National Institutes of Health, Bethesda, USA; 9 Qu Biologics, Burnaby, Canada; 10 UC, San Diego, San Diego, USA; 11 AntiCancer Inc., San Diego, USA; 12 University of Texas, Austin, USA; 13 Center for Biologics Evaluation and Research, US Food and Drug Administration, Silver Spring, USA; 14 Duke University, Durham, USA; 15 Salspera, LLC, Oakdale, USA; 16 Leidos Biomedical Research, Inc., Frederick, USA; 17 University of Minnesota, Minneapolis, USA; 18 Yale University, New Haven, USA; 19 University of Pittsburgh, Pittsburgh, USA; 20 BioMed Valley Discoveries, Inc., Kansas City, USA; 21 Johns Hopkins University, Baltimore, USA; 22 Center for Immunotherapy, Vaccines and Virotherapy , Biodesign Institute, Arizona State University, 727 E Tyler Street, Room A330E, Tempe, AZ 85281 USA

## Abstract

In this White Paper, we discuss the current state of microbial cancer therapy. This paper resulted from a meeting (‘Microbial Based Cancer Therapy’) at the US National Cancer Institute in the summer of 2017. Here, we define ‘Microbial Therapy’ to include both oncolytic viral therapy and bacterial anticancer therapy. Both of these fields exploit tumor-specific infectious microbes to treat cancer, have similar mechanisms of action, and are facing similar challenges to commercialization. We designed this paper to nucleate this growing field of microbial therapeutics and increase interactions between researchers in it and related fields. The authors of this paper include many primary researchers in this field. In this paper, we discuss the potential, status and opportunities for microbial therapy as well as strategies attempted to date and important questions that need to be addressed. The main areas that we think will have the greatest impact are immune stimulation, control of efficacy, control of delivery, and safety. There is much excitement about the potential of this field to treat currently intractable cancer. Much of the potential exists because these therapies utilize unique mechanisms of action, difficult to achieve with other biological or small molecule drugs. By better understanding and controlling these mechanisms, we will create new therapies that will become integral components of cancer care.

## Preamble

The fields of oncolytic virotherapy and bacterial therapy both exploit tumor-specific infectious microbes to treat cancer. As both disciplines have evolved into greater maturity, there has been an increasing appreciation that they share many features in common, to the point where a more consolidated approach (i.e. “oncolytic microbial therapy”) could be fruitful. For example, both modalities rely upon the ability of the therapeutic microbe to selectively infect and kill cancer cells *in situ*, and thereby stimulate a more robust anti-cancer immune engagement in addition to the mobilized cellular and humoral responses that clear the microbe. This White Paper is designed to increase the interactions between these growing fields of microbial oncolytics, and introduce their advances to the wider community of scientists and clinicians working on immunotherapies for cancer. We also describe the state of the field of microbial cancer therapy and point the direction where development and greater synergies with other fields are needed to increase the number of patients and indications that could benefit from this powerful modality.

## Potential of microbial anti-cancer therapy and prevention

Microbial therapy has had several prominent successes in recent years, including the commercialization of the first licensed virotherapeutic, Imlygic (T-VEC), and numerous viral and bacterial candidates progressing through clinical trials. The approval of Imlygic has heralded a renewed interest in microbial therapies. There are many common issues between virus- and bacteria-based therapies and addressing them concurrently will help advance and unify these two research communities. Specifically, we will spell out the major limitations still facing microbial therapies and what steps are necessary to promote greater translation into clinical use, especially in combination with other emerging modalities such as cancer immunotherapy.

Microbial therapies (viral or bacterial) are derived from naturally occurring microorganisms that have often been genetically modified to reduce systemic pathogenicity and increase anti-cancer efficacy. Microbial therapies eliminate malignant tissue by various mechanisms, such as *in situ* production of cytolytic or immunostimulatory agents within tumor beds. Both viral and bacterial therapies have been shown to be capable of re-sensitizing tumors that have suppressed immune surveillance within the tumor microenvironment. Stimulation of the immune system amplifies the desired anti-tumor responses, clearing distant tumor tissue and preventing recrudescence of the cancer.

The potential to cure cancer, or render some as chronically treatable, is the force that is driving discovery and innovation with microbial therapies. Microbial therapies have the potential to provide solutions to many clinical needs that cannot be addressed by current cancer therapeutics. These needs include treatment of refractory metastatic cancers, multidrug-resistant cancers, and cancers that evade immune clearance. This group affects almost all cancer sites including solid carcinomas of the digestive and reproductive systems, melanoma, sarcoma, and blood cancers. Due to the diversity and plasticity of candidate microbes, the development of multiple microbial therapies has the unique potential to address each of these widespread problems. Continual improvements in genetic manipulations have greatly sped up the time from idea generation and preclinical proof-of-concept to clinical testing, and these increases can only be expected to continue to improve in the future.

An essential strength of microbial-based therapies is their specific targeting of cancerous cells and tissues. As such, microbial therapies are well-suited as therapies for metastatic disease, the primary cause of death from cancer. In addition, microbial therapies can circumvent the multi-drug resistant phenotypes that inevitably limit the long-term effectiveness of targeted small-molecule therapies. Microbial therapies have been created to reverse the immune suppression frequently exhibited in late stage tumors that is essential for the proliferation of many cancers [1]; and motile bacteria or carrier-cell loaded viruses have been developed to penetrate tumorous regions inaccessible to standard biologics and small molecules [2]. These factors allow for potent tumoricidal effects capable of generating secondary immunotherapeutic effects while simultaneously minimizing off-target tissue damage and reducing local and systemic toxicities.

Microbiota can be manipulated, and these manipulations can be designed to offer features that may improve their ability to treat cancer. For example, the expression of microbial genes can be controlled by features of the environment, such as hypoxia, or other features of cancer cells, such as abnormal gene expression. Recombinant microbiota incorporating genetic features that are turned on or off in the presence of cancer-specific changes may enable a very large dose-response index, improving safety while enhancing the anticancer effects [3].

Strategies for prevention with microbes is less developed than therapeutic strategies. Microbes that have been envisioned as cancer preventatives include prophylactic vaccines that target precancerous lesions and viruses that enhance and prime the immune response against precancerous cells. Microbial prophylactic vaccines have the potential to target cancers with viral etiology.

## History of microbial therapy

Microbial therapy against cancer has a long history. The interaction of bacteria and cancer has been known for several centuries. Records go back at least 200 years describing cancer patients going into remission after a bacterial infection [4]. For example, in 1867, the German physician Busch reported that a cancer went into remission when the patient contracted erysipelas, now known as *Streptococcus pyogenes* [4]. This strategy was adopted and promoted by William B. Coley of New York Cancer Hospital, which later became Memorial Sloan-Kettering Cancer Center. Coley read about 47 cases of cancer where each patient became infected with bacteria and tumors regressed. In response, Coley looked for evidence in his hospital and located two patients whose tumors regressed after *S. pyogenes* infection. He then began treating patients and had excellent results infecting cancer patients with *S. pyogenes*. His first patient recovered from head and neck cancer. Coley subsequently used killed *S. pyogenes* in combination with a second killed organism now known as *Serratia marcescen* bacteria [5, 6]. The mixture of the killed organisms became known as Coley’s Toxins [5, 6]. James Ewing, for whom the Ewing sarcoma is named was Coley’s boss, did not allow Coley to continue to use his toxins. Coley died deeply disappointed in 1936 and thus ended bacterial therapy of cancer for almost 70 years.

Hoption Cann et al. [7] compared the outcome of Coley’s bacterial treatment to current chemotherapy and found the 10-year survival rates of Coley’s patients, whose records were meticulously maintained by his daughter, Helen Coley Nauts, were comparable to patients receiving current conventional therapy [7]. Coley is now known as the “father of immunotherapy of cancer”, and bacterial therapy is thought by many to be exclusively immunotherapy. However, a large number of studies have shown that, in addition to being immunological, bacteria can be engineered to directly kill cancer cells [4, 8–10].

The history of virotherapy for cancer is similar to bacterial therapy [11, 12]. Viruses were discovered later than bacteria and were only determined to be of a particulate nature in 1917 [13]. However, it had been observed that viral infection could lead to tumor regression. In 1904, Dock reported the regression of a patient’s leukemia after infection with influenza [14, 15]. There has been interest in viral therapy ever since the appearance of these early reports. It wasn’t until 1949 that a trial was attempted after it was observed that hepatitis had an effect against Hodgkin’s disease [16]. It would take several more decades and the advent of genetic engineering before viruses could be made effectively nontoxic, as shown by the seminal work of Martuza, who showed that herpes simplex virus could be used to treat glioma [17].

## Current status of microbial therapy: Major preclinical and clinical advances

The past few decades have seen an increase of systematic development and testing of microbial therapies. Enthusiasm for continued development of such therapies is due to early successes in preclinical and clinical studies. A number of viruses from diverse genera have been developed into anticancer therapies, several genera of bacteria have been tested in preclinical studies, and a few bacterial platforms have been tested in clinical settings [18, 19]. These rationale-based scientific investigations into microbial therapies led to the approval of the first-in-class, oncolytic-herpes-simplex-virus-1-derived therapy, Imylgic [20] by the US Food and Drug Administration (FDA) and European Medicines Agency (EMA) in 2015 for the treatment of late stage melanoma (see Case Box #1). These oncolytic viral therapies demonstrated an exceptional safety profile through Phase III clinical trials.

Although initially designed as tumor lysing agents, the oncolytic virus (OV) platform also possesses activity as anti-vascular agents, gene therapy vectors, and their therapeutic capacity is intrinsically linked to their immune-stimulatory capabilities [21, 22]. The major developments in the field have focused on enhancing their capacity as oncolytic vaccines, and as disruptors of the immune-inhibitory tumor microenvironment. This focus has been particularly important in the clinic, where the development of correlative assays as surrogate markers for efficacy and combinatorial strategies with other anti-cancer agents reflect the capacity of oncolytic viruses as unique immunomodulators [23].

For bacteria-based microbial therapies, many preclinical studies have shown retarded tumor growth and increased survival [24, 25]. Complete tumor regression was achieved with oncolytic bacteria in immunocompetent animals bearing syngeneic tumors and in companion dogs with spontaneous tumors [10, 26, 27]. Bacterial strains have been engineered with therapeutic payloads that resulted in enhanced antitumor activity [18]. Preclinical pharmacologic and toxicologic studies have been conducted to support bacterial strains used in clinical studies. In general, properly attenuated live bacteria have satisfactory safety profiles in both healthy and tumor-bearing animals [28, 29]. Following intravenous administration, attenuated bacteria or bacterial spores are cleared from the circulation and the clearance organs, i.e. liver and spleen, in hours to several days [2, 28]. Recent clinical trials showed encouraging response rates with dually attenuated *Listeria monocytogenes* designed to deliver recombinantly expressed tumor-specific antigens and the bacterium was well tolerated [30–35].

A prime example of a current cancer therapy based on an attenuated microbe is the treatment of superficial bladder cancer with the Bacillus Calmette-Guerin (BCG) vaccine. This modality likely functions by the nonspecific stimulation of immune responses against the tumor, and represents the only bacterial-based cancer therapy that is currently an established medical standard-of-care [36].

## Unmet medical needs: Opportunities for microbial therapy and prevention

Novel treatment strategies are urgently needed to fill the unmet medical needs for cancer patients where the four traditional categories of cancer management are not effective. These pillars of cancer therapy include surgery, radiation, chemotherapy and more recently, immunotherapy [37]. Recent success with immune-checkpoint blockade (ICB) monotherapy has led to FDA approval and use to control many cancer types [37–40]. However, many patients don’t respond to ICB agents because of (1) intrinsic genetic characteristics of the cancer; (2) an immunosuppressive microenvironment; (3) host environmental influences, or (4) insufficient tumor antigen load to stimulate effective anti-cancer cellular responses [41, 42]. For example, patients with advanced pancreatic ductal adenocarcinoma, metastatic prostate cancer, or mismatch repair-proficient (MMRp or MSI-h) colorectal cancer generally fail to respond to anti-CTLA-4 or anti-PD1/PD-L1 monotherapy and continue to pose therapeutic challenges to oncologists [43–48]. In addition to primary resistance to ICB, acquired resistance to ICB has also been reported in both animal models and in patients, which contributes to treatment failure and disease recurrence [49–52]. The mechanisms of acquired resistance to ICB include, but are not limited to: (1) loss of tumor-suppressor gene, such as PTEN [51, 53] or overexpression of β-catenin [52], (2) up-regulation of inhibitory molecules such as PD-L1 and VISTA [50], (3) loss of neoantigens in the evolved tumors [49], (4) epigenetic silencing of chemokines that facilitate tumor-specific T cell recruitment to tumors [53], (5) loss of B2M required for cell surface expression of HLA-I [54, 55], and (6) loss-of-function mutations in JAK1 or JAK2 [54].

Other examples of refractory cancers are melanoma, urothelial carcinoma, some lung cancers and malignant pleural mesothelioma (MPM). Two forms of melanoma that lack effective treatment are locally-advanced unresectable disease that is refractory to immune-checkpoint inhibitors, and refractory visceral metastatic disease. Similarly, in urothelial carcinoma, non-muscle invasive bladder cancer can be refractory to Bacillus Calmette-Guerin (BCG) and locally advanced muscle invasive and/or metastatic disease can be refractory to both platinum agents and ICB. In lung cancer, treatments are needed for patients who have failed chemotherapy and are PD-L1^low^ and PD-L1^neg^, indicating poor response to ICB agents targeting the PD-1/PD-L1 axis. The poor efficacy of standard therapy for MPM (surgery, chemotherapy and radiation) would be improved by the addition of new modalities. Unmet needs that could be addressed with microbial preventive agents include cancers with viral etiology such as hepatitis C virus (HCV), Merkel cell polyomavirus (MCV), Epstein-Barr virus (EBV), human herpesvirus 8 (HHV-8), and human T-cell lymphotropic virus type 1 (HTLV-1). While many other opportunities for microbial oncology therapeutics exist, they are most likely to get clinical traction in indications such as these examples where high-priority unmet needs exist. If durable clinical benefit is obtained in these indications, microbial-based therapies would be well positioned to address other oncology indications.

## Microbial strategies attempted to date

Microbial therapies have unique mechanisms to control cancer. It is the uniqueness of these mechanisms that provides the potential for these therapies to address unmet needs that cannot be attained with molecular therapies. There are three major mechanisms employed by microbial therapies to control cancer: gene delivery, immune stimulation, and direct oncolysis. For many viral therapies, these three mechanisms are intimately linked and are driven by single genes or single mechanisms. For bacteria, which are larger and contain more genetic material, these mechanisms can be integrated or built from disparate mechanisms.

### Virotherapy and cancer vaccines

Most virus-based therapies are multi-mechanistic. These therapies function by delivering genes into the cancer host cells. Many of these delivered genes are designed to either directly engage the immune system or to lyse cancer cells. This lysis leads to the release of cancer specific antigens that in turn stimulate the acquired immune system. Virus therapies can be grouped into two types: cancer vaccines and virotherapies. Vaccines encode and express cancer-specific antigens or epitopes. In contrast, virotherapies do not encode exogenous tumor antigens but drive lytic mechanisms that kill cells to unmask or unleash endogenous antigens from the cells that become infected. “Oncolytic vaccines” are a hybrid strategy, in which an oncolytic vector also encodes a tumor antigen or neo-epitope.

Viral therapies can utilize either replicating or non-replicating viruses. Replication-competent viruses retain the ability to replicate and spread to adjacent un-infected tumor cells and are, in theory, dose amplifying. Eventual death of virus-infected tumor cells ensues a second mode of efficacy resulting from host anti-tumor immune responses roused by the release of tumor antigens along with inflammation due to infection. Together this has the potential to educate a host memory response to clear both injected lesions as well as un-infected metastatic disease.

Most nonreplicating viruses are utilized predominantly as gene delivery vehicles that can deliver toxins, tumor suppressors, genes that result in drug sensitization to chemotherapy, or genes that convert a non-toxic drug into its toxic intermediate [56]. These agents have been exploited as carriers of tumor suppressor genes, such as p53, or to deliver cytotoxic suicide genes. This has the potential to be a self-limiting mechanism because tumor cell death results in clearance of the vector [57]. An example of immune-modulating gene therapy that just entered first-in-human trials is an adenovirus encoding for human FlT3L to elicit an antitumor immunity [58]. There are several ongoing clinical trials with non-replicating viruses (Table 1).

**Table 1:** Examples of recent trials for non-replicating-virus-mediated cancer gene therapy

Biological Agent	Virus (gene)	NCT#	Indication
AAV2hAQP1	AAV (Aquaporin-1)	NCT02446249	Squamous cell Head and Neck cancer
NP2	HSV-1 (NP2)	NCT00804076	Cancer pain
Ad5CMV-p53	Adenovirus (p53)	NCT00003147	Liver cancer
TK99UN	Adenovirus (HSV TK)	NCT00844623	Hepatocellular carcinoma

Many viral strains have been exploited for anti-cancer drug development. DNA viruses include: Adenoviridae, HSV-1, Parvoviridae (see Case Box #2), and Poxviridae. RNA viruses exploited for development into tumor agents of destruction include Paramyxoviridae, Picornaviridae, Reoviridae, Retroviridae, and Rhabdoviridae [59]. Some strains of viruses that are not associated with human pathology, such as the normally rabbit-specific myxoma virus, have also shown a natural propensity for replication and destruction in human tumors. A table of current active clinical trials with different oncolytic viruses is shown in Table 2.

**Table 2:** Examples of active studies with oncolytic viruses

Biological Agent	Virus	NCT#	Indication
TG6002	Vaccinia virus	NCT03294486	Glioblastoma
ADV/HSV-tk	Adenovirus	NCT03004183	NSCLC and triple-negative Breast Cancer
Pexa-Vec	Vaccinia	NCT03206073 NCT02562755	Colorectal Hepatocellular Carcinoma
LOAd703	Adenovirus	NCT02705196	Pancreatic Cancer
CG0070	Adenovirus	NCT02365818	Bladder cancer
MV-NIS	Measles	NCT00408590 NCT02192775	Ovarian cancer Multiple myeloma
HF-10	HSV-1	NCT03252808	Melanoma
GL-ONC1	Vaccinia virus	NCT02714374 NCT02759588	Solid Tumors Ovarian cancer
VCN-01	Adenovirus	NCT02045602	Advanced Solid Tumors
Ad-MAGEA3	Adenovirus	NCT02879760	NSCLC
OBP-301	Adenovirus	NCT03190824	Unresectable Metastatic Melanoma
G207	HSV-1	NCT02457845	Pediatric Brain Tumors

Abbreviations: *NSCLC* non-small cell lung cancer

### Cancer vaccination: Gene delivery to engage the immune system

Viral vectors are the default selection for gene-delivery therapies. Research over the past three decades has led to a deep understanding of viral tropism, genome sequences, regulatory elements and immune-evasion mechanisms. These efforts have culminated in the development of a versatile arsenal of increasingly tumor-selective viral-vector technologies. Replacement of naturally-occurring viral genetic-regulatory elements with tumor-specific elements has been a key enabling feature that has led to the generation of numerous tumor-selective viral vectors [60, 61]. These modified viruses limit gene expression and infectivity to tumor cells while sparing normal cells, allowing for the incorporation of a wide array of different genes encoding for potent effector proteins such as cytokines, chemokines, protein toxins, gene silencing RNAs, micro RNAs, and prodrug-converting enzymes [62]. Viruses have been used as vaccination agents, either by inducing cross-reactive immunity, or more commonly being engineered to express specific antigens to be targeted by adaptive immune responses.

To enhance the immune response, the processing of specific antigens can be altered. For example, gene sequences encoding fusion proteins linked to intracellular-trafficking elements have been demonstrated to improve and enhance antigen presentation [63, 64]. The potential to locally augment an adaptive immune response to tumor neoantigens is quite promising, as there is a consensus that shared mutated antigens are uncommon, and the majority of mutated antigens serving as neoantigens are private. Consequently, local secretion of a variety of immune-stimulating molecules, such as GM-CSF and IL-12 have been engineered into microbes, as well as adaptor proteins such as MyD88 [65, 66].

Another approach to cancer vaccination is “in situ” vaccination (ISV). Vaccines exploit antigens to be recognized by the immune system and adjuvants to activate the immune system, but for cancer, the problematic issue has been to choose exactly which antigens are the “right” ones. ISV uses the tumor as the source of the antigen and injects the adjuvant directly into the tumor. The overall goal is to generate a strong local antitumor response that reverses the local tumor-mediated immunosuppression and then to have that local response become systemic to fight potential or recognized metastatic disease [67, 68]. For over 30 years, the standard-of-care to treat superficial bladder cancer after surgery has been applying Bacillus Calmette-Guerin to the bladder lumen as an ISV immune adjuvant [69]. Another current ISV treatment in clinical usage is resiquimod, a TLR 7 agonist, for cutaneous T cell lymphoma treatment [70]. There are a variety of ongoing studies investigating the best approaches to *in situ* vaccination, such as developing a plant virus, cowpea mosaic viral-like nanoparticle, as a potent ISV reagent [71].

### Oncolytic viruses: Vaccines and virotherapy

One limitation of cancer vaccines is that, although tumor-associated antigens have been well characterized, the presence of tumor-specific neoantigens generated by somatic mutations within the genome of a cancer cell, are most commonly private, with shared antigens representing a very small percentage of known antigens [72]. Consequently, strategies to identify and generate tumor-specific immune response are being sought. To create immunogenic viral vectors, natural viruses have been modified to express tumor-specific antigens during the tumor-cell-lysis portion of the viral-infection cycle. This modification results in *in situ* “oncolytic vaccination”, at least partly promoted by antigen cross-presentation [73]. To better promote the immune response against these host antigens, several oncolytic viruses encode for immunostimulatory cytokines such as Type-I interferons or GM-CSF [73]. An alternative approach to strict viral-mediated oncolysis is tumor-selective dissemination and expression of a prodrug-converting enzyme followed by administration of the prodrug (e.g. Toca 511/FC) [74]. There are numerous active clinical trials with oncolytic viruses (Table 2).

One advantage of recombinant viruses that are oncolytic, is that they are capable of causing tumor cytoreduction, but also capable to elicit adaptive immune responses to the antigens, including neoantigens released by tissue injury and death induced by the viral life cycle. Immune activation by oncolytic-virus infection of tumor tissue comprises both, immediate effects of innate immunity and also adaptive responses for long-term tumor-specific activity. Much of the enthusiasm surrounding oncolytic vaccines revolves around their appeal as multi-mechanism therapeutics [21]. Oncolytic viruses are proposed to function through a combination of (1) selective killing via tumor-specific cell lysis (oncolysis) and (2) activation of innate and adaptive immunity via immunogenic cell death, immunostimulation from recognition of viral constituents, and release of both viral and tumor antigens [75]. As mentioned, oncolytic viruses can also be engineered to encode transgenes to further engage the host immune response.

This approach to microbial-based cancer therapy may be particularly well suited for use in indications not considered to be highly immunogenic, but where overexpression of specific tumor-associated antigens could be of value. These recombinant microbes are engineered to deliver or express tumor associated antigens in professional and immature antigen-presenting cells *in vivo* to break peripheral tolerance of tumor-antigen-specific effector cells. Measurable clinical responses to microbial therapies designed for this purpose have been achieved and, while response rates are low, they are significant, appear to be durable, and highlight the promise of this approach.

### Bacterial therapy

Similar to viral therapies, bacterial therapies can function predominantly by direct oncolysis or by engaging the immune system. Direct oncolysis can be mediated by secreting exotoxins or competing for nutrients [76]. Alternately, intracellular bacteria can kill host tumor cells by inducing apoptosis or by uncontrolled proliferation resulting in bursting of the infected tumor cells [77]. In addition, bacterial infections inevitably activate both innate and adaptive arms of the immune system, which target not only the bacteria but also tumor cells [78].

An essential strength of bacterial therapies is their specific targeting of cancerous cells and tissues. Each modality targets in a distinct way. For example, active bacteria have been engineered to only colonize the microenvironment of tumors [79, 80]; oncolytic bacteria have been engineered to induce cell death specifically in cancer [81]; and immune-sensitizing bacteria have been engineered to induce responses to cancer-specific antigens either directly [30] or indirectly through epitope spreading [82]. Because of these varying targeting mechanisms, microbial therapies are well-suited as therapies for metastatic disease.

A large number of bacterial genera have been investigated as potential therapeutics (Table 3). The bacterial genera receiving the most attention thus far include *Listeria monocytogenes* (Case Box #3), *Clostridium novyi* (Case Box #4)*,* and *Salmonella* largely because our understanding of their pathogenicity, physiology, and genetics has led to well defined attenuation strategies critical for safe use of live bacteria in patients*.* Each genus of bacteria is better suited to a specific mechanism. The predominant organism for immune engagement is Listeria because of its propensity to invade immune cells. Facultative enterics, like Salmonella and Escherichia, have been used for both purposes because of their ability to stimulate the innate immune system and because of their genetic plasticity. Obligate anaerobes, such as Clostridium, can localize in hypoxic regions of tumors, a characteristic that could circumvent adverse features associated with resistance to radiation therapy or chemotherapy [83].

**Table 3:** Bacterial genera investigated as anti-cancer therapeutics

Genus	Species
*Clostridium*	*C. histolyticum, C. sporogenes, C. novyi, C. acetobutylicum, C. beijerinckii*
*Salmonella*	*S. enterica*
*Listeria*	*L. monocytogenes*
*Bifidobacterium*	*B. adolescentis, B. longum*
*Lactobacilli*	*L. acidophilus*
*Escherichia*	*E. coli*

### Immune engagement with bacteria

The intrinsic ability to stimulate the innate immune system makes bacterial-based cancer vaccination strategies an attractive approach. Many genera of bacteria accumulate in the tumor microenvironment and once there can convert the tumor microenvironment (TME) through proinflammatory responses. Tumor-colonizing bacteria alter the immune suppressive environment of tumors and make them immune stimulating. This conversion can be achieved through different approaches, such as (1) incorporation or cloning of adjuvants into the bacteria, (2) cloning highly immunogenic antigens as an alternative for neo-antigens, (3) encoding with immunogenic cytokines which evoke anti-tumor immune responses, (4) cloning of checkpoint inhibitors into the bacteria, or (5) depletion of immune suppressing macrophages or myeloid-derived suppressor cells (MDCS). Species that have been investigated for this approach include attenuated *Listeria monocytogenes*, *Salmonella enterica*, and *Clostridium novyi*-NT. All of these bacteria selectively survive and multiply in the TME. For example, attenuated *Listeria monocytogenes* have been designed to deliver recombinantly-expressed tumor-specific antigens [84]. Similarly, strains of attenuated *Salmonella enterica* have been engineered to secrete biologically-active cytokines such as IL-2 [18, 85].

Many classes of microbial anticancer therapies rely upon induction of immunogenic cell death (ICD) to propagate effective anti-tumor immunity. ICD is recognized as a critical process that initiates the tumor-immunity cycle and ultimately leads to an adaptive immune response [86]. In contrast to tolerogenic cell death and other forms of tumor cell demise, ICD is characterized by three distinct molecular events which are necessary to prime and activate tumor-infiltrating dendritic cells: (1) translocation of calreticulin from the endoplasmic reticulum to the cell surface (i.e. ecto-calreticulin), (2) passive extracellular release of high-mobility-group box 1 (HMGB1) from the nucleus, and (3) extracellular release of ATP [87, 88]. These molecular “danger” signals collectively function as damage-associated molecular patterns (DAMPs) which recruit professional antigen-presenting cells into the TME which can then uptake tumor-associated antigens released from dying tumor cells.

Bacterial-based vectors naturally stimulate the innate system through activation of multiple immune pathways. Diversity among membrane components between bacterial species manifests in differential expression and presence of structurally-conserved pathogen-associated molecular patterns (PAMPs). These molecules include flagella, pili, adhesins, lipotechoic acid and lipopolysaccharide and each activates specific toll-like-receptor (TLR) family members to elicit distinct innate immune-signaling cascades that ultimately translate to a comprehensive immune signature unique to each bacterial organism [89]. Different bacteria engage the innate immune system in distinct ways including repolarization of tumor associated macrophages (TAMs) from an immunosuppressive pro-tumor M2 phenotype to a M1 anti-tumor phenotype; selective elimination of both peripheral and tumor-associated myeloid derived suppressor cells (MDSCs); and promotion of dendritic-cell maturation at the tumor site [90]. CD8^+^ T cells are a major player in the adaptive antitumor immune response [26, 91]. A bacterial infection establishes an inflammatory environment that sensitizes CD8^+^ T cells to low-density tumor antigens by enhancing proximal T-cell receptor (TCR) signaling [92].

### Direct tumor destruction with bacteria

In addition to their intrinsic anti-cancer effects, bacterial vectors can be genetically engineered or modified to express proteins with direct tumoricidal properties [18]. Because of their ability to host large amount of foreign DNA, bacteria can be used as a platform for synthetic biology and the construction of entire functional genetic circuits [93]. Bacteria have the capacity to accommodate heterologous DNA of larger than 300 kb [94]. The possibility of delivering ubiquitously-toxic molecules relies on the bacterial ability to selectively colonize, proliferate and express genes only in tumors [95]. This specificity is maintained by introducing stable genetic circuits that are selectively responsive to unique qualities of the TME, such as differential pH, nutrient and/or oxygen availability [96, 97]. To further limit toxicity, quorum-sensing systems have been developed that focus expression to tumors and prevent expression in normal tissue [98].

The most prominent bacterial toxins exploited as therapeutics are pore-forming cytolysins derived from *Escherichia coli* [99, 100], or *Staphylococcus aureus* [101, 102]. These molecules are natively expressed by bacteria, easily secreted, and induce apoptosis in mammalian cells [102–104]. They are secreted as monomers that form multimeric pores after incorporation into the membranes of eukaryotic cells [105–107]. Several cytotoxic cytokines that have been explored are FAS ligand (FASL), TNF-related apoptosis-inducing ligand (TRAIL) and TNFα [25, 108–111]. In addition to being immunogenic, these molecules directly induce apoptosis [25] and are more toxic to cancer cells than normal cells [25, 108]. As therapeutics, TNFα and TRAIL are effective against bladder, breast, colon, glial, lung, ovarian, pancreatic, prostate, and renal cancer cells [25, 112]. However, these cytokines cannot be administered systemically because they would induce significant toxicity. Localized production by bacteria colonized within tumors obviates this problem and focuses treatment on the cancerous lesion.

Immune-cell-targeting bacteria, such as Listeria monocytogenes, can reduce tumor mass by delivering radioactivity. For example, 188-rhenium has been coupled to Listeria through anti-Listeria antibodies and shown to be effective against mice with pancreatic cancer [113, 114]. Bacteria can also be engineered to deliver siRNA. To achieve this strategy, *E. coli* has been transformed to transcribe shRNAs from a plasmid containing the invasin gene *inv* and the listeriolysin O gene *hlyA*. These two genes encode bacterial factors that are needed for transfer of shRNAs into tumor xenografts [115].

### Combination of viruses and bacteria with other modalities

The greatest therapeutic strides with oncolytic platforms are likely to be achieved for diverse patients through multipronged pharmacological and immunomodulatory approaches. One approach is to identify chemotherapy drugs that can alter cellular signaling to sensitize tumor cells to viral replication and/or burst. Cellular DNA damage, after induction by radiation or chemotherapy, can augment HSV-1 replication by amplifying viral burst [116]. Based on these observations, G207 has been evaluated for safety and efficacy in combination with radiation for adult and pediatric patients with brain tumors [117]. Another rationale for utilizing high dose chemotherapy has been to use the lympho-depletive effects to counter innate immune cell mediated virus clearance. Encouraging preclinical results from this approach have resulted in a clinical trial (NCT00450814) wherein safety and efficacy of measles virus-NIS has been tested as a stand-alone therapy and in combination with cyclophosphamide. While chemotherapy is known to have immune suppressive effects, manipulating the timing of chemotherapy with viral agents can also exploit the development of anti-tumor immunity due to the release of chemotherapy-induced lymphopenia during the time of infection and hence tumor antigen presentation. This allows for expansion of tumor-selective T cells to maximize antitumor benefits [118, 119].

The greatest attention has been paid to combination with checkpoint-inhibitory antibodies [120–124]. The recent success of immune-checkpoint blockade with humanized antibodies blocking negative regulatory receptors such as CTLA-4 and PD-1 has brought forth renewed interest in enhancing virus mediated anti-tumor immunity in conjunction with these agents. A randomized open Phase II study evaluating the combination of talimogene laherparepvec with ipilimumab (a humanized anti-CTLA-4 antibody therapy) in patients with advanced melanoma revealed a greater antitumor activity of the combination without increasing adverse events [125]. The combination of T-VEC and anti-PD-1 immunotherapy modulates the TME and promotes intratumoral T-cell infiltration resulting in overall and complete responses among patients with advanced melanoma [126]. Oncolytic viruses have been shown to recruit and provide cytokine support to CAR-T cells [127–129]. Many of these strategies have demonstrated a favorable toxicity profile, high overall-response rates, and marked intra-tumoral T cell infiltration and anti-tumor T cell responses [121, 126].

While many bacterial-based therapeutics impart significant single agent activity, combination with other modalities may increase efficacy. Bacterial proliferation is often specific to the immune-privileged necrotic/hypoxic regions of solid tumors [130, 131], sparing the well-perfused regions that will eventually grow back. Traditional cytotoxic therapies, such as chemotherapy and radiation therapy, are most effective against cells in those well-perfused regions. Combining bacteria with these cytotoxic therapies generates synergistic effects [24, 132, 133]. Another strategy employed in combination with bacteria is the expression of cleaving enzymes that activate prodrugs specifically in the TME [134]. For example, delivery of HSV-TK with Salmonella converts gancyclovir to its toxic form and results in a dose-dependent reduction in tumor burden [135]. Similarly, Listeria has been used to deliver prodrug-converting enzymes like purine-nucleoside phosphorylase and a fusion protein consisting of yeast cytosine deaminase and uracil phosphoribosyl transferase [136].

### Non-living bacterial strategies

Non-living bacteria-based therapies are alternative strategies that have several advantages over living organisms. This category of therapies includes bacterial minicells, cell-wall complexes, outer-membrane vesicles, and other bacterial derived therapies [137–139]. Bacterial minicells are small chromosomeless bacterial particles that contain all the natural and recombinant components of their parent cells yet are unable to divide and are non-infectious. In addition to being as amenable to recombinant engineering as their parental progenitors (i.e. can be engineered to carry oncolytic proteins, for example), minicells have limited metabolic activity and can be stably loaded with small-molecule cytotoxic drugs by passive diffusion.

Site-specific immunomodutors (SSIs) are composed of components of specific bacterial pathogens. When administered subcutaneously, SSIs result in a recruitment and activation of innate immune cells (including macrophages and NK cells) in the organ in which the bacterial pathogens, from which they are derived, commonly causes infection. For example, QBKPN, derived from a lung pathogen (*Klebsiella*), stimulates a lung-specific anti-cancer immune response, including macrophage recruitment and M1 polarization, NK cell recruitment and activation, and upregulation of the NKG2D pathway [140]. QBKPN reduces lung-cancer burden in mouse models and downregulates PD-1 and PD-L1 systemically in lung-cancer patients [140].

### Prevention with microbial therapies

Microbial therapies have the potential to prevent cancer by several distinct dimensions. First, microbiota can be manipulated to destroy tissue, which can be utilized as a form of cancer prevention. Microbial treatment of a neoplastic lesion may serve not only to eradicate the lesion, but also stimulate an immune response. That response may, in turn, prevent subsequent invasive cancer. Cancer prevention by tissue ablation forms the basis for many contemporary cancer prevention strategies targeting particularly high-risk individuals, including prophylactic mastectomy, oophorectomy, colectomy, and thyroidectomy for significant reduction in the incidence of subsequent breast cancer, ovarian cancer, colon, and thyroid cancer, respectively [141]. A second preventative approach would be manipulation of the microbial pathogens that drive tumorigenesis and are associated with an altered composition of commensal microbiota (dysbiosis). Microbes are estimated to be involved in 15% to 20% of cancer cases. Preclinical studies demonstrate that modulation of inflammation, DNA damage, and metabolite production are potential mechanisms of oncogenesis or tumor suppression, which may be altered with changing the microbial composition [142]. A third approach would utilize microbial prophylactic vaccines to target cancers with viral etiology, similar to HPV and HBV vaccines [143–145]. A fourth strategy would be enhancement of the immune system response by modifying dendritic cells (DCs) to improve vaccine potency.

## Lessons learned from clinical studies

### Lessons from trials with oncolytic viral therapies

In the last two decades, numerous clinical trials have been initiated with oncolytic viruses. These trials have taught many lessons about the mechanisms of cancer vaccination and how to select the most appropriate patients (see the Mayo Clinic Experience with Virotherapy, Case Box 5). For example, experience with cancer vaccines has demonstrated that targeting tumor-specific antigens alone is likely insufficient to generate effective anti-tumor immunity and to prevent immune escape. Optimal T-cell responses come from not only the engagement of the T-cell receptor binding to the MHC-peptide complex (signal 1) but also to a second signal with cytokine stimulation or a costimulatory molecule (signal 2). Two FDA-approved cancer vaccines highlight this concept. Sipuleucel-T (Provenge), an autologous dendritic cell vaccine contains a fusion protein (PA2024) consisting of the target antigen (prostatic acid phosphatase) as well as immunostimulatory GM-CSF, which can augment dendritic cell activity and maturation. Similarly, T-VEC produces GM-CSF to enhance immunogenicity [146].

An intriguing story has emerged suggesting that effective cancer immunotherapy can promote “antigen spread” [137]. The notion of antigen spread suggests that immunogenic cell death of target antigen-expressing tumor cells release non-targeted antigens that prime subsequent adaptive immune responses against non-targeted antigens. This phenomenon has been observed among patients treated with PROSTVAC, which is a prostate cancer vaccine that contains transgenes for prostate-specific antigen (PSA) and three T-cell co-stimulatory transgenes: B7-1 (CD80), leukocyte function-associated antigen-3 (LFA-3), and intracellular-adhesion molecule-1 (ICAM-1) [138, 139]. This observation suggests that T-cells specific for tumor-antigens not present in the initial vaccine construct may be an important additional mechanism of action and potentially indicative of a favorable immune response.

Numerous studies investigating the role of innate anti-viral defense responses in ocolytic viral therapy have uncovered that rapid virus clearance minimizes the initial burst of tumor cells and thus limits virotherapy by negatively regulating virus lytic tumor destruction [147]. While infection-induced host responses can clear the virus, this initial inflammatory response to viral infection is also thought to be an essential step leading to the influx of antigen presenting cells and the development of subsequent anti-tumor immunity [148]. The Yin-Yang balance between innate virus clearance limiting therapeutic index and the stimulation of host anti-tumor immunity has emerged as a heavily debated subject, wherein host innate-defense responses are touted as being both beneficial and detrimental to therapy. Most likely the situation is further compounded by variables that impact host response such as the virus platforms used, different tumor types and stages, TME, and prior-treatment status of the patient.

### Lessons from trials with bacterial therapies

Results from preclinical and clinical studies suggest that tumor-targeting bacteria may be a useful option for cancer treatment (see Case Box #6). However, toxicity will be a major challenge for their clinical development. A successful outcome may be achieved only when toxicities associated with the therapeutic infection can be effectively managed without compromising the therapeutic effect. Despite the complex safety considerations, the FDA has allowed several clinical trials with tumor-targeting bacteria and bacterial vaccines designed to elicit a systemic response (Table 4) [10, 149–152]. Clinical studies on companion dogs with spontaneous tumors have also been conducted [10, 153, 154]. The tumor-targeting bacterial strains tested in both canine and human studies showed reasonable safety profiles in general and promising antitumor activities in some studies. It has become clear even with the limited clinical studies that substantial bacterial colonization is required for any significant clinical benefit. Therefore, careful selection of patients with tumors likely to provide an optimal niche for bacterial colonization will be important in the design of clinical studies. As tumor necrosis/hypoxia is a critical prerequisite for colonization of some bacteria, tumor biopsy or noninvasive companion diagnostic approaches based on angiography and hypoxia/necrosis imaging may help define such a patient population [155–157].

**Table 4:** Examples of active clinical studies with bacteria

Biological Agent	Bacterium	NCT#	Indication
IL-2	Salmonella	NCT01099631	Liver Cancer
*C. novyi-NT*	Clostridium	NCT00358397 NCT01118819 NCT01924689	Solid Tumors
APS001F	Bifidobacterium	NCT01562626	Solid Tumors
JNJ-64041757	Listeria	NCT02592967 NCT02625857	NSCLC Prostate Cancer
JNJ-757	Listeria	NCT03371381	Lung Cancer
pLADD	Listeria	NCT03189030	Metastatic Colorectal Cancer
ADU-623	Listeria	NCT01967758	Astrocytic Tumors
ADXS11-001	Listeria	NCT01598792 NCT01266460	Oropharyngeal Cancer Cervical Cancer
CRS-207	Listeria	NCT01675765 NCT00585845 NCT02575807	Malignant Pleural Mesothelioma Adenocarcinoma of the Pancreas Ovarian Cancer
GVAX & CRS-207	Listeria/GVAX	NCT02004262	Pancreatic Cancer

In these clinical trials, Listeria do not colonize the tumor microenvironment

Because of the potential toxicity of these therapies, knowing when and how to intervene are critical issues which will have direct impact on clinical outcomes. Dose determination is another important issue with the clinical development of bacterial therapy. Dose escalation is a standard practice in Phase I clinical trials. However, the effective dose for live bacteria depends more on the target tumor tissue where they can multiply than on the administered dose. A large tumor with extensive necrosis and minimal infiltration of inflammatory cells is more likely to support a robust bacterial infection, resulting in pronounced tumor response and toxicity, regardless of the administered dose. In contrast, a small tumor or a tumor with minimal hypoxic/necrotic regions is unlikely to show significant response even if a large number of bacteria are administered.

## Important scientific questions

To fully capture the potential of microbial therapy it would be beneficial to describe the design space as comprehensively as possible. An optimal microorganism must (1) target only tumor cells, (2) have tolerable toxicities or side effects, and (3) be generically stable. These organisms would act by (1) engaging the immune system, (2) reducing tumor-associated immunosuppression, or (3) delivering anticancer agents (e.g. interleukins, sh-RNAs, and toxins like Listeriolysin O or α-haemolysin, which would cause cell lysis). Targeting could be enhanced by generating microorganisms that (1) selectively infect poorly vascularized tissues not accessible to drugs; (2) specifically target precancerous cells, metastatic tumors, and/or dormant cancer cells; (3) target tumor neovascularization; or (4) have organ-specific affinity (e.g. respiratory viruses). Microbial cancer therapy could be effective beyond direct treatment as (1) preventatives and vaccines, or (2) by preventing tumor recurrence.

To achieve these goals, many questions must be addressed about the interactions of the microbes with tumors and tumor cells. Each of the following questions is a furtive research area that, when addressed, will be instrumental in the development of effective microbial anticancer therapies.

- What mechanisms control (1) microbial tropism to tumors and metastases, (2) invasion and microbe-mediated lysis of tumor cells, (3) microbe-initiated immune responses, and (4) processing and presentation of microbe-delivered or tumor-cell derived antigens?
- How can toxicities and deleterious side effects be managed, eliminated or prevented?
- What mechanisms can be introduced into bacteria and viruses to increase genetic stability?
- Can the immune system be selectively evaded to limit microbial clearance and inactivation?
- Can new targets be identified for microbial recognition of tumor cells?
- What is the optimal balance between microbe-initiated immune stimulation, microbial attenuation, and immune evasion?
- Are there cancer types particularly sensitive or resistant to microbial therapies? What biological characteristics make a tumor more sensitive or resistant to a microbial therapy?
- Can microorganisms prevent the transformation of precancerous cells into tumor cells?
- Can microorganisms eliminate residual cancer cells after treatment?
- Can companion diagnostics be used to predict the response to microbial therapeutics?

Most of the questions about the mechanisms of microbial therapies can be classified into four categories: simulation of the immune system, control of toxicity, control of delivery, and safety.

### Immune stimulation and engagement

To date, the complex effects of a viral tumor infection on the TME and the consequences for the tumor-infiltrating immune cell compartment are poorly understood. There is growing evidence that a tumor infection by an oncolytic virus opens up a number of options for further immunomodulating interventions such as systemic chemotherapy, generic immunostimulating strategies, dendritic cell-based vaccines, and antigenic libraries to further support clinical efficacy of oncolytic virotherapy [126]. More specifically, can microbial infection of an immunologically “cold” tumor (i.e. low mutational load and/or nonresponsive to immune checkpoint inhibitor monotherapy) induce conversion into a “hotter” milieu where novel endogenous tumor epitopes are now presented more efficiently to the immune system?

Novel approaches are needed to alter the immunosuppressive microenvironment of tumors and facilitate the recognition of tumor antigens. The lack of immune-cell infiltrates within tumors negatively correlates with the response to immunogenic cell death (ICB) and patient survival [52, 158]. Improved understanding of the critical role and mechanisms of Batf3 dendritic cell antigen uptake and cross-presentation may yield novel insights to optimize dendritic cell-based vaccine strategies. Because innate immune responses against infection and malignancy utilize similar evolutionarily conserved pathways (i.e. cGAS-STING pathway) microbial anticancer agents may be particularly effective at engaging and amplifying anti-tumor immunity [159]. Batf3-dependent CD103^+^/CD8α^+^ dendritic cells (DCs) are critical for cross-presenting tumor antigens in tumor draining lymph nodes (TDLNs). Cross presentation allows for priming and expansion of naïve CD8^+^ T cells that recognize the cognate antigen [52, 160–162]. Possible strategies to expand CD103^+^/CD8α^+^ DCs include systemic administration of FMS-like tyrosine kinase 3 ligand (FLT3L) or intratumoral delivery of immune adjuvants such as TLR3, TLR7, and STING agonists.

Bacterial infections of tumors naturally stimulate an immune response. However, it is not entirely clear how intratumoral infections enhance antitumor immune responses. It has been suggested that the antitumor immune responses result from the infection-induced immunogenic immune-cell death causing epitope spreading [84]. A complex immunotherapeutic strategy is needed for a sustained and robust anti-tumor effect. It is not enough to administer immunostimulatory cytokines, such as IL-2 and IL-15, to elicit innate and adaptive anti-tumor immune responses, primarily involving NK cells and cytotoxic T-cells, respectively. It is now understood that tumors avoid immune-mediated elimination by the secretion of immunoinhibitory molecules, recruitment of immunosuppressive cells, and the upregulation of immunoinhibitory checkpoints such as CTLA-4 and PD-L1 [163]. Studies addressing the questions of how bacteria kill tumor cells are still limited. Additional mechanisms will surely be revealed once data from more studies on this issue become available.

### Control of efficacy

One of the major questions affecting bacterial therapy is how to produce a high enough local concentration of a therapeutic. Compared to viruses, bacteria are larger and can be used to deliver multiple gene products. However, bacteria are small relative to mammalian cells (about 1000-fold by volume), meaning that any produced protein must be effective at low concentrations. Regardless of the mode of action, efficacy depends on the local concentration of the therapeutic molecule. A high local titer can be achieved by manipulation of bacterial gene expression, metabolism, or protein secretion. The local tumor concentration could also be enhanced by increasing tumor colonization and proliferation. Better understanding of these mechanisms could greatly improve bacterial therapy.

In comparison, viral therapeutics have limited coding capacity due to small genomes and are limited to delivering only a small number of genes. In the context of delivering an immunomodulatory gene sequence, most viral vectors deliver a single gene (e.g. GM-CSF, IL-12, or IFN-α2b) and conflicting data raise questions as to whether this is sufficient for immune activation robust enough to break peripheral tolerance to tumor antigens. Strategies to address this may include addition of other immunomodulators (e.g. TLR agonists such as imiquimod) to therapeutic regimens, and/or use of viral vectors encoding regulatory RNA sequences implicated in the global regulation of immunosuppression or pathways at the level of the transcriptome.

### Control of colonization and delivery

The control of localization and delivery is essential for bacterial therapies. Focusing therapy specifically to malignant tissue can greatly increase efficacy and reduce toxicity to normal tissue. Early clinical trials with bacteria showed that facultative anaerobes colonize human tumors, but at lower densities and with more heterogeneity than in mouse models [150]. Although much is known about the mechanisms that control bacterial tumor colonization, more strategies are needed to harness these mechanisms and make the organisms perform optimally in human tumors.

There are three categories of bacterial therapies based on how they accumulate in tumors: obligate anaerobes (e.g. Clostridium and Bifidobacterium), facultative anaerobes (e.g. Salmonella and Escherichia); and immune-cell-targeting bacteria (e.g. Listeria). The spores of obligate anaerobes germinate exclusively within the hypoxic regions of the cancer [10, 26, 164]. Vegetative anaerobes cannot survive in oxygenate environments of normal organs and can only survive in the anoxic regions of tumors [130, 165]. Facultative anaerobes accumulate in tumors via several interacting mechanisms. After intravenous injection, bacteria flood into tumors following inflammation [166] and become entrapped in the vasculature [167]. Small molecules produced by tumors draw bacteria into the tissue, where they preferentially replicate in the favorable microenvironment [79, 168]. Listeria accumulates in tumors by infecting (MDSC) [113, 169, 170]. These cells are present in blood in large numbers of patients and mice with cancer [171], and are selectively attracted to the TME [172]. Once at the tumor site, Listeria spreads from MDSC into tumor cells by polymerization of actin filaments and production of the pore-forming enzyme listeriolysin O [173]. For all three types of bacteria, immune suppression prevents clearance in the tumor environment but not in normal tissues, which lack immune suppression [174].

#### Delivery of viral particles

Because viral particles are inert outside of host cells, classic delivery of naked virus depends on passive accumulation. High viral titers can be safely administered when using a highly tumor-selective virus and tumors with leaky vasculature. To be effective, the inoculum needs to remain in the circulation for sufficient time to allow passage through the tumor vascular bed. This becomes a particular issue for viral species that have not evolved to spread in the blood, and so have little or no protection against complement or removal by the reticuloendothelial system [175, 176]. Even species that are well adapted to evade these innate immune responses are likely to be severely restricted in their ability to be delivered systemically when faced with an anti-viral adaptive immune response, especially circulating neutralizing antibodies. This might be due to previous vaccinations (e.g. vaccinia, measles), prior exposure to a virus (e.g. adenovirus) or simply as a result of repeated treatment with the same viral therapy.

The easiest way to avoid such delivery hurdles is to directly inject the virus into the tumor. Intratumoral administration would circumvent antiviral immune responses, enable more direct contact with cancer cells and reduce systemic toxicity [177]. However, this method of administration creates another set of issues, including the need for a surgeon to perform such procedures; an inability to target multiple metastases; reaching areas of the tumor beyond the needle tract; and ensuring the virus is not delivered to necrotic regions or simply escapes the tumor.

Several strategies have been developed to try to enhance the systemic delivery of oncolytic viral therapies. One approach has been to chemically modify the viral particle itself [178], such as chemical attachment to large inert molecules (notably polyethylene glycol, PEG) [179–181] or wrapping of the viral particle in a synthetic lipid envelope [182] or polymer coat [183]. Other strategies include complement inhibition [184] and cytokine conditioning to mobilize a cellular population upon which the virus can hitchhike [185]. These approaches have successfully demonstrated the ability to de-target viruses from non-tumor cells (notably hepatocytes) and to increase circulation time. However, they often suffer from an inability to reliably release infectious virus in the tumor. Because the modifications often inhibit the natural routes of viral cell entry, they need to be selectively removed within the tumor or alternative cell entry routes developed. Similar approaches involving enzymatically removing immune activating surface molecules (such as through deglycosylation) [186] or genetic engineering of the virus itself (such as swapping of coat proteins between related viral strains that have evolved to spread differently in a host) [187] have demonstrated greater successes.

Another strategy that has demonstrated success in pre-clinical models is to pre-infect *ex vivo* cells that are known to traffic to the sites of tumor beds following infusion into the patient. In this way, cells (known as “carrier cells” or “Trojan horses”), including some stem cells or immune cell populations that naturally traffic to and infiltrate tumors, can be used as delivery vehicles to actively carry the viral payload to the tumor target [188]. This has the additional advantages that the virus may be concealed from innate or adaptive immunity effectors in the circulation and may replicate within the carrier cell, so further increasing viral delivery into the tumor. However, there are also additional complexities, such as ensuring cell trafficking is not inhibited by viral infection, defining doses and manufacturing primary cells or cell lines for clinical use. In addition, the cells that are most commonly attracted to tumors tend to be involved in immunosuppression or disease progression, so care must be taken to ensure they cannot provide pro-tumor properties. However, it has also been demonstrated that some viruses naturally infect lymphocytes in circulation and utilize these cells as *in situ* carrier vehicles to enhance spread within a host. These phenomena have also been shown to occur in the clinic resulting in enhanced delivery of oncolytic viruses, such as reovirus [189]. As such, there is potential to further exploit these naturally occurring pathways to enhance systemic delivery of oncolytic viruses in general.

### Safety

Modern microbial therapies have an excellent safety record. In almost all oncolytic virus trials to date, few adverse events have been attributed to replicating viruses. However, off-target effects and viral mutation/transmission remain ongoing concerns. An open question is the optimal administration of oncolytic viruses. When replicating viruses are administered systemically, non-malignant cells are spared because they retain more robust anti-viral defenses than transformed cells. Infected normal cells either destroy the oncolytic virus or commit apoptotic suicide resulting in a self-limiting infection. As demonstrated by intralesional administration of T-VEC, distant tumor regression suggests that *in situ* vaccination has primed a systemic immune response (the abscopal effect). It remains unclear if systemic administration can improve response rates without additive or synergistic toxicity. Because human bodies have evolved to fight infections, immunity to microbial agents may develop in response to systemic administration. Major challenges in the development of systemic delivery are serum neutralization and hepatotoxicity. A neutralizing antibody response has been observed in almost every patient treated with viruses, but has not been correlated with a response or lack thereof.

Numerous tumor-targeting bacterial strains have shown a therapeutic benefit in experimental tumor models for decades, and yet clinical development has been slow to follow. Safety is a major concern for therapy with live bacteria, where therapeutic effect is intertwined with toxicity. Preclinical pharmacologic and toxicologic studies with live bacteria have shown satisfactory safety profiles in both healthy and tumor-bearing experimental animals [28, 29]. However, robust intratumoral colonization is required for optimal therapeutic effect. Such an aggressive infection, even confined within the target tumor, will inevitably result in clinical symptoms and laboratory findings that may be considered dose-limiting toxicities (DLTs). This dilemma is perhaps best illustrated in the therapy with oncolytic bacteria. The objective for oncolytic bacterial therapy is essentially to convert a solid tumor into an abscess that forms when an extensive bacterial colonization and subsequent tumor lysis take place [10, 190]. Abscess formation is obviously a desired therapeutic effect and a significant toxicity at the same time. Therefore, whether or not the abscess should be qualified as a DLT is debatable. In this way, it is the very mechanism of tissue-specific pathogenesis that provides the potency to be exploited as a curative. Because of the plasticity of bacteria, the overarching question is whether clever genetic manipulations can be made to utilize endogenous mechanisms and shift the balance toward an effective therapy with minimal safety concerns [98].

## Where next?

Three issues that are essential for the development of microbial therapies are 1) finding the most appropriate predictive animal models, 2) development microbial therapies into cancer prophylactics, and 3) defining standard techniques for GMP manufacture.

### Better animal models

Animal models are invaluable to the understanding of neoplastic development and to the evaluation of novel therapeutics. Each model has its benefits and deficiencies which should be carefully considered prior to the study. The implementation of multiple models over the course of the translational development path will aid in recapitulating as many aspects of human biology as possible. The choice of model should balance cost and efficiency with accurate representation of pathology and progression.

Allo- and xenotransplant models provide time-saving experimental protocols and less expensive animals than more complicated models. The use of immunocompromised xenotransplant models can provide information about the efficacy of oncolytic therapies, but are inappropriate for immunotherapies. Immune-deficient mice lack the ability to develop an adaptive memory response against both the microbe and the implanted tumor. Although allo-transplant models are immunocompetent, the speed of tumor growth in transplant models can prevent development of systemic or TME immune states present in human cancer [191].

Genetically engineered mouse models (GEMMs) may more acurately recapitulate essential aspects of human cancer [192]. Autochthonous tumors form in GEMMs from a single cell and grow with a time course similar to human cancer. This slower rate allows more natural structural maturation of the TME [193] and more closely approximate the molecular and histopathological features of human neoplasms [194, 195]. The major limitations of autochthonous tumors in GEMMs are their cost, relatively low mutational load, and reduced clonal heterogeneity. Many GEMMs result in numerous transformed “cancer-seeding” niches, with uneven synchronies but relatively homogeneous genetics, which can make GEMMs hard to cure via microbial therapies.

Patient-derived xenografts (PDX), reconstitute the heterogeneity of the genomic aberrations seen in human cancers. This property overcomes the major limitation of GEMMs, but introduces additional limitations. In this model, primary human tumor tissue is introduced into “humanized” mice that have been reconstituted with a human hematopoietic system [196]. This model can be cost prohibitive and has several limitations. There are inherent deficiencies of the human immune system when it is developed within a mouse, and tumor cells do not completely interact with host stromal and immune compartments in the local microenvironment. This system is also limited by the technical challenge of human leukocyte antigen (HLA) matching implanted tumors with the immune system of the reconstituted mice.

The use of rodents to determine the safety and efficacy of novel agents has inherent limitations because infectious biological agents have co-evolved with mammals and are species-specific pathogens. The inherent resistance of mice to some human pathogens makes preclinical testing of these microbial agents particularly challenging. Alternate models to evaluate the safety and/or efficacy are Syrian hamsters and non-human primates.

Evaluating therapeutic efficacy in veterinary clinical trials is also a useful approach as it permits safety and efficacy testing in large spontaneous tumor models [197–202]. Spontaneous canine and feline cancers represent a naturally occurring heterogeneous model of cancer that resemble human disease in their histopathological and molecular characteristics [203]. Two important aspects of companion animals are that (1) the microbiome of pet dogs shares many features with that of humans [204–207], and (2) the process of immunoediting occurs over the course of months to years, a time frame not achievable in murine models [208]. When considering a large companion animal trial, it is important to consider the frequency and biology of tumors which can sometimes be quite distinct from human disease.

### New strategies for microbial-based cancer prevention

Microbial therapies have the potential to be potent cancer prophylactics. To be effective, prophylactic vaccines could (1) target cancers with viral etiology, (2) directly target precancerous lesions, or (3) enhance the immune response against precancerous cells. Some cancers have a viral etiology including liver cancer, cervical, head and neck, anal cancer, Burkitt Lymphoma, nasopharyngeal carcinoma, Merkel Cell Carcinoma, Kaposi Sarcoma, and HTLV-1 associated T-Cell Leukemia/Lymphoma. While the effectiveness of vaccination against cancers with viral etiology has been demonstrated for human papillomavirus (HPV) and hepatitis B (HBV), other vaccines are needed to target additional viruses that are highly associated with cancer, including HCV, MCV, EBV, HHV-8, and HTLV-1. Microbial autologous DNA/RNA vaccines could prevent recurrence by targeting known tumor-associated antigens. Targeting precancerous lesions would prevent the transformation into tumor cells. These vaccines could be prepared to target antigens from patient-derived tumors or human tumor cell lines. Prophylactic vaccines might be useful in cases where there is known family history risk, including known genetic determinants that lead to a high risk of cancer. Microbial-based therapies could also prevent cancer by eliminating cancer-associated microorganisms, or by targeting neovascularization and dormant cancer cells. Microorganisms that target tumor antigens would also be useful for eliminating residual cancer cells after treatment.

### GMP manufacture

The GMP manufacture of microbial-based cell therapies is a regulated process that centers on product safety, consistency, and stability. Any investigational new drug (IND) application submitted to the United States Food and Drug Administration (FDA) is required to have a comprehensive description of the chemistry, manufacturing, and control (CMC) information used while generating the product. The FDA has recently published detailed information, in the form of an industry guidance, on the information that must be submitted in an IND pertaining to bacterial- or viral-based biologic products [209].

Critical to the CMC package for any biological product manufactured under GMP conditions is the ability to properly characterize the sterility, purity, identity, potency, and stability of the drug substance and drug product, including its raw materials, and critical intermediates. GMP manufacture of microbial-based cell therapies begins with raw materials and a list of each component used in the manufacturing process. Second to raw materials is the generation of a seed stock, expansion and characterization of the microbial Master Cell Bank (MCB), and any further expansion into a working cell bank (WCB). Key considerations in the manufacture of the seed stock, MCB and WCB include verification of the absence of any lysogenic prophage (for bacteria), genome sequence information characterizing any chromosomal modifications (deletions and additions), phenotypic confirmation of attenuation, microbial purity (clonality), cell viability, and the stability, restriction digest pattern(s), and sequence identity of any episomal constructs (e.g. bacterial expression plasmids). The stability of these features within the MCB and WCB should be characterized over time to gain an understanding of when failure occurs. During early phase production, a master manufacturing record (MMR) is established based on at least one process development experience at the intended manufacturing scale. The MMR is subject to industry standard Quality Assurance (QA) and Quality Control (QC) review procedures and sponsor approval before being accepted for use and employed to officially codify and record manufacturing steps.

Manufacturing processes are typically divided into upstream, downstream, fill/finish, and release testing components. For bacterial cell therapies, the upstream process usually begins with MCB/WCB vial thaw and includes fermentation and formation of the crude cell paste. Downstream processing of bacterial cell therapies terminates after formulation and that process intermediate is defined as the formulated drug substance. A suite of release tests is typically performed on the formulated drug substance prior to advancing to the fill/finish stage. In fill/finish, the drug substance is individually vialed, packaged, labeled as appropriate and is now defined as the drug product. Vialed drug product is subject to further release testing and released for stability testing and use in clinical trials. For non-living bacteria-based products and virotherapies, sterility must also be determined prior to use. With living bacterial cell therapies, demonstrable microbial purity is required.

#### NCI translational research assistance

The National Cancer Institute (NCI) offers translational research assistance for microbial-based cancer therapies through programs such as the NCI Experimental Therapeutics (NExT) program (Table 5). The NExT program provides drug discovery and development resources and expertise to advance promising new therapies to clinical development. Following a peer-reviewed application process, biotechnology-based therapeutic products are supported by an array of contract resources. Intellectual property (IP) brought in by applicants is maintained by the originator. Any new IP developed under the contract is offered to the applicant under exclusive license.

**Table 5:** Examples of translational research support at NIH

Program	Mission/Objective	Website link
NCI: Experimental Therapeutics (NExT) Program	Supports promising new anticancer-drug (small molecule, biologics) discovery and development projects towards clinical evaluation and registration.	https://next.cancer.gov/
NCI: Division of Cancer Prevention (PREVENT)	Supports development in cancer prevention intervention and biomarkers.	https://prevention.cancer.gov/major-programs/prevent-cancerpreclinical
NCATS: Bridging Interventional Development Gaps (BrIDGs) Program	Advances promising therapeutic agents for both common and rare diseases through late-stage preclinical development toward an IND application.	https://ncats.nih.gov/bridgs
NCATS: Therapeutics for Rare and Neglected Diseases (TRND) Program	Supports preclinical development of therapeutic drugs to treat rare and neglected disorders toward the goal of an IND application.	https://ncats.nih.gov/trnd
NIAID: Translation Resource Tools	Supports preclinical and clinical research for vaccines, diagnostics, and therapeutics.	https://www.niaid.nih.gov/research/therapeutic-developmentservices
NINDS: Create Bio Program	Supports optimization of biotechnology product and biologics-based therapies for development and IND-enabling studies, as well as early-phase clinical trials.	https://www.ninds.nih.gov/Current-Research/Research-Funded-NINDS/Translational-Research/CREATE-BIO
NHLBI: SMARTT Program	Accelerates translation of research for therapeutic candidates or diagnostic imaging agents from in vivo efficacy to IND submission for treatment of heart, lung, or blood diseases.	https://www.nhlbismartt.org/

The Frederick National Laboratory for Cancer Research’s (FNLCR) Biopharmaceutical Development Program (BDP) is one such contractor used for development of clinical grade biotechnology products. Operated by Leidos Biomedical Research Inc., the BDP facilities include development laboratories, QC analytical laboratories, and GMP facilities, totaling approximately 60,000 sq. ft. The production areas within the GMP facility are designed to ISO 5 (Class 100), ISO 7 (Class 10,000) and/or ISO 8 (Class 100,000) specifications to achieve levels appropriate for GMP bulk biologics manufacturing and final filling operations. The BDP includes capabilities in process development, GMP manufacturing, analytical testing, QA oversight, and regulatory strategy (https://frederick.cancer.gov/Science/Bdp/Default.aspx). Over the past 15 years the BDP has provided NCI with expertise in developing biologic products such as recombinant proteins, monoclonal antibodies, immunotoxins, cell banks, immunocytokines, vaccines, and mammalian viruses. BDP technical expertise includes bacterial fermentation, mammalian cell culture, protein and virus production and purification, and aseptic fill/finish manufacturing operations. Analytical capabilities include protein biochemistry, bioanalytical testing, physio-chemical analysis, virus testing, molecular biology analysis, stability assessments, and standard quality control techniques for raw materials and facility operations. The BDP manufacturing capabilities and regulatory support are most appropriate for IND-enabling and clinical use. Once a commercial partner is identified, BDP transfers the technology to the company for commercial manufacturing and licensing.

In addition to the NExT program, translational research resources are available through other NCI and NIH programs. These programs provide access to drug development resources to enable submission of IND applications to the FDA. Some examples of NIH translational research programs are listed in Table 5, with website links for further details and application instructions.

## Guidance for future development: Regulatory perspectives

The development of new microbial therapies is regulated by federal regulatory bodies (e.g., the US FDA) to assure the safety and rights of human subjects for initial clinical trials. They also ensure that the marketing approval of these therapies are based on the demonstration of safety and substantial evidence of efficacy. At least three regulatory issues need to be considered for developing microbial therapies: unintended spreading (shedding) of therapeutic organisms, demonstration of systemic effect of oncolytic viruses, and potential infection following treatment with bacteria-based therapies.

Microbial-based therapies often involve live viruses or bacteria that can replicate and thus self-amplify. Thus, one safety concern from a regulatory perspective is that these microbes may be shed from patients and spread to individuals who are in close contact. Although genetic modification may limit the ability of these microbes to be pathogenic, shedding could still occur, particularly for microbial agents that are systemically administered. Therefore, early in the clinical development of microbe-based therapy, it is important that the clinical-trial design includes plans to monitor shedding. Such monitoring not only serves to protect the public health but also provides information for the design of later-phase trials. The details of the monitoring program such as sample collection, monitoring methods, cut-off thresholds and their interpretation, may vary among different microbes. FDA has published a Guidance for Industry to address these issues entitled ‘Design and Analysis of Shedding Studies for Virus or Bacteria-Based Gene Therapy and Oncolytic Products’ [210]. This guidance can help sponsors and developers to design appropriate shedding studies.

One of the most challenging issues is how to assess the efficacy of intratumorally administered oncolytic viral therapy (IT OVT), a local therapy, in the context of a systemic disease such as metastasis. To address this issue, it is crucial to consider whether such an IT OVT could lead to a systemic treatment effect. The measures to take to demonstrate such an effect could include 1) observation of an abscopal-like effect showing tumor regressions in the lesions that are not injected, in particular, visceral lesions for an OV given to non-visceral lesions; 2) demonstration of prolongation of progression-free or overall survival in patients who have received an IT OVT compared with those who have received control; and 3) demonstration of a systemic immune response. However, the latter could only be used as supportive if it is not accompanied by an improvement in clinical outcome assessment.

One of the main concerns of bacteria-based cancer therapy is that these bacteria could cause clinically significant infection and sepsis, especially in cancer patients who may be already immunocompromised due to their underlying disease and/or from concomitant treatment. Some invasive procedures may be needed to administer some of these live bacterial products which may add additional risks to patients. Thus, in early clinical trials design, investigators and sponsors need to consider appropriate plans to mitigate these concerns (e.g., antibiotic use post administration of live bacterial products).

## Conclusion

We hope that this White Paper will serve to nucleate the growing field of oncolytic microbial therapy. There is much excitement about the potential of this field to treat currently-intractable cancers, and to synergize with other growing modalities such as immunotherapy. In the process of putting this paper together, we saw how many parallels exist between bacterial and viral therapies. We also learned that they both face similar obstacles to commercialization. By harnessing a broad set of mechanisms that cannot be achieved with other strategies, microbial therapies (both viruses and bacteria) have the potential to create the next generation of therapeutics and will accelerate our gains against cancer mortality. Of the many factors that generate enthusiasm about microbial therapies, the ability to specifically target malignant tissue and deliver genetically-engineered payloads are two of the greatest. Achieving these visions will require integration across many disciplines, including oncology, infectious disease, immunology, surgery, manufacturing, preclinical modeling, and regulatory affairs. By providing therapies with unique mechanisms of action, microbial therapies are destined to become integral components of effective cancer management.

## Box 1 Case examples: Talimogene laherparepvec (T-VEC, Imlygic)

T-VEC is a modified herpesvirus with deletion of two viral genes and insertion of GM-CSF. The initial developer of T-VEC was BioVex Ltd, a UK-based company which originated from the academic lab of Robert Coffin at University College London in 1999. It was designed to induce a more systemic benefit than previous oncolytic viruses by providing an immune enhancement component. To increase local anti-cancer effects T-VEC was derived from a higher potency clinical isolate of HSV than had been used before. Two viral genes were deleted: ICP34.5 and ICP47. Deletion of ICP34.5 provides tumor selectivity and deletion of ICP47 leads to increased expression of US11 and usually inhibits antigen presentation. Increased US11 partially overcomes the phenotype of ICP34.5 deletion, which reduces tumor replication, without reducing tumor selectivity. The gene encoding GM-CSF was inserted into the virus to recruit and activate dendritic cells. This was based on the prior observation that tumor-cell-derived cancer vaccines expressing GM-CSF (so-called GVAX vaccines; Cell Genesys) had demonstrated clinical promise. Previous GVAX vaccines needed to be derived from tumor biopsies (i.e. be autologous), which was logistically challenging. T-VEC was intended to generate an autologous GVAX-like vaccine directly in the patient *in situ*, without the patient-specific manufacturing complexity, and with the direct tumor-killing ability of an oncolytic virus.

T-VEC initially began a phase 1 study in the UK in 2003. This study demonstrated that the optimal dosing strategy was a lower initial dose followed by higher subsequent doses. The initial dose served to seroconvert or boost anti-HSV immunity, which then reduced the side effects for subsequent higher doses. These side effects were transient fevers and local reactions. Using this dosing approach T-VEC was well tolerated, and showed promising signs of anti-tumor activity, including necrosis and shrinkage of injected tumors and tumors which were nearby, and inflammation of distant, uninjected tumors [211]. Further studies were conducted in head and neck cancer in combination with chemotherapy and radiation [212], pancreatic cancer (unpublished), and melanoma [213]. The 50 patient melanoma study showed a 26% response rate in a mixed population of melanoma patients (Stage IIIc-IVM1c, both previously treated and first line), which was felt very promising (particularly as at that time - 2007- no drug had demonstrated improved survival in melanoma) and led to the initiation of a phase 3 study.

The Phase 3 study [20] was conducted in the UK, US, Canada and South Africa, and enrolled 436 Stage IIIb-IVM1c patients randomized 2:1 to be treated with T-VEC or subcutaneous GM-CSF. The primary endpoint agreed with the FDA under SPA was 'durable response rate' (DRR). This provided a rigorous assessment of efficacy and required that patients maintained a continuous six month period of response (PR or CR) to count towards the primary objective of the trial. This was also based on the assessment of all tumors rather than only a subset of tumors, and allowed that progression could occur before response, i.e. as might be expected with an immune-based approach. This study was initiated by BioVex in 2009, and then completed by Amgen, by whom BioVex was acquired in 2011. The results of this study showed a 16.3% DRR for T-VEC vs 2.1% for GM-CSF, with an overall response rate (ORR) of 26.4% vs. 5.7%, respectively [20, 210]. Median survival was 23.3 months for T-VEC and 18.9 months for GM-CSF (*P*=0.051). The improvement in survival was greater in patients without visceral disease (Stage IIIb, IIIc or IVM1a disease) and in previously untreated patients. The trial met its primary objective in the ITT population, and was approved by the FDA for that patient group in 2015. However, the most appropriate patients for treatment with single T-VEC appeared to be those without visceral disease or who were first line. EMA approval was received for these groups in 2016.

## Box 2 Case examples: Rodent protoparvoviruses

Parvoviruses are the smallest viruses currently being developed as oncotherapeutic agents. The rodent protoparvovirus H-1 is currently in Phase I/IIA clinical trials against glioblastoma [214, 215] and pancreatic cancer [216] in Germany. Viral capsids are non-enveloped and extremely rugged, simplifying their production, purification and storage [217–219], as well as enhancing their tissue penetration properties. The protoparvoviruses belong to the same virus family as the adeno-associated viruses (AAV), which have already found extensive clinical use as gene therapy vectors. Unlike AAV, protoparvoviruses, do not require a helper virus for vegetative growth. However the rodent protoparvoviruses can infect human cells only if the cells are neoplastically transformed, and thus are intrinsically oncotropic [220, 221]. They exhibit a relatively high spontaneous mutation rate in cell culture, allowing them to be rapidly selected for enhanced targeting of tumor cells by serial passage [222]. A replicating, but non-propagating, vector system has been developed from these viruses, capable of expressing up to two immunomodulatory genes in place of the viral-coat-protein coding sequence. The vector construct can be packaged into a tumor-cell-target-enhanced capsid selected in vitro. The dual-transgene can express either co-stimulatory or immunoregulatory molecules, or both, from the same cassette. Viral infection induces robust pro-phagocytic signals, such as ectopic calreticulin expression, and secretion of alarmins, such as HMGB1. Current applications using parvoviruses include glioblastoma multiforme, malignant melanoma and pancreatic ductal adenocarcinoma.

## Box 3 Case Examples: Listeria

Listeria activates the NAPH-oxidase pathway resulting in high levels of reactive oxygen species (ROS) that induce immunogenic tumor cell death [223, 224]. When Listeria was used to deliver natural-killer (NKT) cell-activator alphagalactosylceramice (αGalCer), NKT cells were recruited to the tumor microenvironment (TME). This recruitment resulted in immune stimulation of CD8 T cells to Mage-b and a nearly complete elimination of metastases [225]. Listeria also converts subpopulations of immune suppressive MDSCs into immune-stimulating myeloid-derived suppressor cells (MDCSs), producing high levels of IL-12 [169]. Listeria infects macrophages (CD11+ cells) in metastases and alters their function to favor immune stimulation [84]. Stimulation of CD8 T cells contributes to the elimination of metastases [84]. Since Listeria infects tumor cells, which may present Listeria antigens on their membrane, it is that suggestive Listeria-specific T cells killed the tumor cells. Listeria also reduce the Treg population in the TME, which correlated with improved efficacy of Listeria-Her2/neu in mice with Her2/neu-expressing cancers [226].

## Box 4 Case examples: *Clostridium novyi*

*Clostridium novyi*-NT (*C. novyi*-NT) is an attenuated strain of *Clostridium novyi*, a spore-forming, Gram-positive, obligate anaerobe that germinates in hypoxic tumor environments. This strain lacks the alpha-toxin gene, which is the major necrotizing toxin responsible for Clostridium species pathogenicity [76, 77]. When administered intravenously (IV) or intratumorally (IT*), C. novyi*-NT spores replicate within the hypoxic regions of tumors, eliciting robust tumor lysis in animals including companion dogs bearing spontaneous solid tumors. The anti-tumor effect of *C. novyi*-NT has also been observed in humans in two clinical trials sponsored by BioMed Valley Discoveries. In a Phase I study of *C. novyi*-NT in patients with advanced solid tumor malignancies, a single IV injection of C. novyi-NT spores was shown to cause tumor destruction in a metastatic lesion of a patient with colon cancer. In another Phase I study it was determined that a single IT injection of *C. novyi*-NT is feasible and it led to significant destruction of injected tumor masses in more than a third of patients treated. The most common toxicities are fever and tumor inflammation, which are expected due to the nature of the therapy. Preliminary findings indicate that *C. novyi*-NT induces a tumor-specific T-cell response**.** Future clinical studies will build on emerging pre-clinical data of the combination of *C. novyi*-NT with immune-checkpoint inhibitors.

It is worth noting that spores of the obligate anaerobic strain *C. novyi*-NT germinated exclusively in hypoxic tumor tissues but not in non-malignant hypoxic lesions [29, 227], underscoring the tumor-targeting specificity of obligate anaerobes. Suggestive evidence of immunogenic tumor cell death through ROS by *C. novyi*-NT has been found as well. Mice cured from CT26 tumors by *C. novyi*-NT were protected from rechallenge with the same tumor cells, indicating underlying immune-based mechanisms [26]. Since *C. novyi*-NT kills tumor cells through ROS, immunogenic tumor cell death may have led to epitope spreading and rejection of CT26 tumors.

## Box 5 Case examples: Mayo clinic experience with virotherapy

Since its establishment in 1997/1998, the Virotherapy Program at Mayo Clinic has carried out multiple clinical trials using adenovirus, measles virus, reovirus and vesicular stomatitis virus (VSV) platforms. As with many such trials at other Institutions, the road to establishing these trials has typically been very long, expensive and full of new learning opportunities. The overwhelming theme, from the hundreds of patients treated so far, has been that virotherapies have largely proved safe. However, there have been exceptions to this – with one patient treated by intra-tumoral injection of a VSV engineered to express IFN-ß dying days after the virus injection. The cause of this most serious of serious adverse events (SEA) is still being investigated. However, it is clear that virus replication in the liver-metastatic tumor of this patient was very robust and tumor lysis syndrome along with possibly liver-toxic levels of IFN-ß may have contributed. Conversely, although rare, the program has also seen individual cases of great therapeutic success. Thus, one patient with advanced multiple myeloma treated with the highest dose of a recombinant measles virus underwent a dramatic regression of her disease which has proved to be durable out to 4 years following a single-shot treatment. As for the SAE at the other end of the spectrum, it is not completely clear whether this impressive success for virotherapy represents the result of pure oncolysis of the measles virus in the myeloma tumors, immune stimulation following such a high dose of systemically delivered virus or a combination of both.

Perhaps the most important lesson that has come out of these hundreds of treated patients and the millions of dollars spent, is that when great successes, or tragedies do happen, it is critical that all such trials are supported by correlative studies put in place to understand the mechanistic basis behind each one. In this light, even a terrible SAE can provide critical information that will help us to advance the field. For example, deep sequencing of a patient’s tumor which supported (potentially life threatening) abnormally high levels of virus replication can lead to a profile of patient selection criteria which may help to avoid such events in the future. Such information may also help us to develop adjuvant treatments which take tumors predicted to be low-level responders (low virus replication) to a more permissive state. In addition, a full and comprehensive immune-monitoring capability will allow investigators to correlate positive, negative or a lack of clinical responses to immune-virotherapy with host responses, including cytokine profiles. Therefore, it is strongly recommended that comprehensive immune, genomic and virological monitoring assays be put in place before trials recruit their first patients. In this way, the hope is that the current scenario – multiple patients treated with low toxicities and only intermittent responses (good or bad) – may soon be replaced by the preferred situation where the majority of enrolled patients respond well as a result of being pre-selected for specific immune-virotherapies based upon a clear knowledge of their likelihood to respond.

## Box 6 Case examples: Saltikva (MDB401)

Saltikva is attenuated Salmonella armed to produce IL2. It differentiates normal environments from the abnormal, typically hypoxic environments found in solid tumors. There is a preference of varying degrees for different tumor types, but also a clear, ten-thousand-fold preference for tumors over organs such as the healthy liver. Taking residence in tumors and replicating there, this strain of Salmonella incites a host immune response and turns the cancer into a bystander to be destroyed. This ultimately leads to destruction of the tumor, and formation of memory to counter the growth of metastases. Saltikva was not envisaged as a monotherapy. Because it is orally bioavailable and safe, it may synergize with targeted therapies or chemotherapies. Veterinary application of MDB401 has been out-licensed and is on track for market introduction in 2018/19. Next steps in the human development program for Saltikva include clinical trials in the first target, sarcoma of adolescents and young adults.
